# Identification and in silico analysis of cattle DExH/D box RNA helicases

**DOI:** 10.1186/s40064-015-1640-0

**Published:** 2016-01-07

**Authors:** Manish Kumar Suthar, Mukul Purva, Sunil Maherchandani, Sudhir Kumar Kashyap

**Affiliations:** Department of Veterinary Microbiology and Biotechnology, Rajasthan University of Veterinary & Animal Sciences, Bikaner, Rajasthan 334001 India

**Keywords:** RNA helicases, DEAD box, Bioinformatics, Bovine, *Bos taurus*

## Abstract

The helicases are motor proteins participating in a range of nucleic acid metabolisms. RNA helicase families are characterized by the presence of conserved motifs. This article reports a comprehensive in silico analysis of *Bos taurus* DExH/D helicase members. Bovine helicases were identified using the helicase domain sequences including 38 DDX (DEAD box) and 16 DHX (DEAH box) members. Signature motifs were used for the validation of these proteins. Putative sub cellular localization and phylogenetic relationship for these RNA helicases were established. Comparative analysis of these proteins with human DDX and DHX members was carried out. These bovine helicase have been assigned putative physiological functions. Present study of cattle DExH/D helicase will provides an invaluable source for the detailed biochemical and physiological research on these members.

## Background

A fundamental cellular action of RNA helicases is to unwind nucleic acid duplexes and thus, they are required for different cellular processes involving RNA. Among these helicases several members perform their functions in pre-mRNA processing and ribosome biogenesis (Linder 2006). The DEAD and DEAH are the subgroups of the DExH/D family (Staley and Guthrie 1998). The DDX code is used for DEAD box and DHX is used for DEAH box. The basis of nomenclature of these DExH/D helicases is the composition of conserved amino acids in their motif II. DEAD-box and DEAH-box, helicases have D-E-A-D (Asp, Glu, Ala and Asp) and D-E-A-H (Asp, Glu, Ala and His) amino acids respectively at this motif. These proteins have role in RNA metabolism viz. transcription, translation, RNA editing and folding, nuclear transport, RNA degradation and RNA-ribosomal complex formations (Linder and Daugeron 2000; Patel and Donmez 2006). These helicases belong to superfamily 2 (SF2) of the six super families in which all the helicases have been classified (Caruthers and McKay 2002; Tanner and Linder 2001). DExD/H-box proteins have been reported from all the living organisms (Umate et al. 2011; Tuteja and Tuteja 2004a, 2004b; Hartung et al. 2000). The core of these enzymes contains two RecA-like domains separated by a short linker. The N-terminal and C-terminal domains are designated as DEAD-domain and helicase domain respectively (Cordin et al. 2006; Pyle 2008). These domains participate in RNA (substrate) binding and ATP hydrolysis. Alignments of the protein sequences obtained from various organisms have revealed nine highly conserved motifs in DEAD-box proteins (Q, I, Ia, Ib, and II–VI) and eight in DEAH-box proteins I, Ia, Ib, II, III, IV, V and VI (Tuteja and Tuteja 2004a, 2004b; Tanner et al. 2003). Among these motifs, motif II (or Walker B motif) along with motif I (or Walker A motif) and Q-motif are necessary for ATP binding and hydrolysis (Tanner et al. 2003) whereas, motifs Ia, Ib, II, IV and V may be involved in RNA binding (Svitkin et al. 2001).

Genome sequencing of variety of organisms have revealed the presence of different numbers of DExH/D helicases. In a genome-wide comparative study 161, 149, 136 and 213 different RNA helicase genes have been identified in *Arabidopsis thaliana*, *Oryza sativa*, *Zea mays* and *Glycine max* respectively (Xu et al. 2013). Also, 31 DEAD and 14 DEAH putative RNA helicases have been reported from human beings (Umate et al. 2011).

Recently, Steimer and Klostermeier summarised involvement of RNA helicases in infection and diseases (Steimer and Klostermeier 2012). For example dysregulation of these helicases has been linked to a wide variety of cancers. In addition, these proteins have a role in the replication of viruses such as Foot and mouth disease virus infection in cattle and HIV virus in human beings. RNA helicases A (DHX9) has been associated with cattle FMD disease (Radi et al. 2012; Lawrence and Rieder 2009). We can reveal prognostic and diagnostic markers and identify potential drug targets by characterizing these helicases.

Cattle are economically important domesticated ungulates. Phylogenetic analysis has shown a distant clad for cattle as compared to humans and rodents (Murphy et al. 2004) and around 800 breeds have been established serving as resource for the genetics of complex traits studies. The genome sequence for domesticated cattle (*Bos taurus*) was assembled and published in 2009 (The Bovine Genome Sequencing and Analysis Consortium 2009). The sequence reveals presence of a minimum 22,000 genes in cattle. In the present study, sequenced cattle genome was used to evaluate the number of DEAD-box and related family proteins which might be present, along with their phylogeny. The composition of these bovine motor proteins have also been analysed. In silico analysis of bovine DExH/D helicases provided the putative role of these proteins in various RNA metabolism processes which might be operating in *Bos taurus*.

## Methods

### Database search and enlistment of RNA helicases

The sequences for DExH/D family members encoded by *Bos taurus* were downloaded from NCBI/BLAST (http://www.ncbi.nlm.nih.gov.nih.gov). Amino acid sequence of eIF4A1 (Swiss-Prot Id-Q3SZ54) was obtained first from Swiss-Prot using the key words eIF4A1 *Bos taurus*. The input sequence so obtained was used in the Cow RefSeq protein database available at NCBI/BLAST home. The cow genome sequences were searched using program BLASTP-Compare protein sequence against ‘BLAST Cow sequences’ resource. Finally tentative lists of DExH/D family members were compiled and all proteins (DExH/D family members) were assigned unique Swiss-Prot IDs, protein names and gene names. After identification of bovine RNA helicases their phylogenetic analysis was carried out along with helicases of other animals of veterinary importance like horse, pig and sheep. For this key words DEAD and DEAH helicase along with animal name were used to download homologs from pig, horse and sheep from Swiss-Prot database for phylogenetic analysis of these DExH/D helicases vis a vis bovine helicases. The amino acid sequences of both families of RNA helicases were aligned and the neighbour-joining method in MEGA 5.0 was applied to examine their evolutionary relationship (Tamura et al. 2011).

Specific sequences of *Bos taurus* were used for BLASTP search against human homologs as described above to compare their homology. Protein sequences were validated by the presence of signature motifs. Predictive molecular weight and isoelectric point for the RNA helicases were calculated from Sequence Manipulating Suite (http://www.bioinformatics.org/sms2/). Protein localization was studied using WoLF PSORT (http://www.genscript.com/psort/wolf_psort.html) program.

### Motif identification and phylogenetic analysis

The signature motifs for the protein family were identified. Protein sequences of DEAD and DEAH members were first aligned using ClustalW2 program available at http://www.ebi.ac.uk/Tools/msa/clustalw2/ and alignment files were downloaded. Conserved motifs in bovine DExH/D were also identified using the MEME suite (version 4.9.1) at meme.nbcr.net/meme/cgi-bin/meme.cgi. Finally list of signature motifs was generated. Phylogenetic analysis was performed using MEGA5 program (http://www.megasoftware.net/) by the Neighbour-Joining method (NJ) with parameters; complete deletion option, p-distance and bootstrapping method with 1000 replicates (Tamura et al. 2011). Final image was obtained using the MEGA5 program. Domain analysis was performed using the program Scan Prosite (http://expasy.org) and these domain structures were used in the figures.

## Results and discussion

### Identification and validation of *Bos taurus* DExH/D family members

**Fig. 1:**
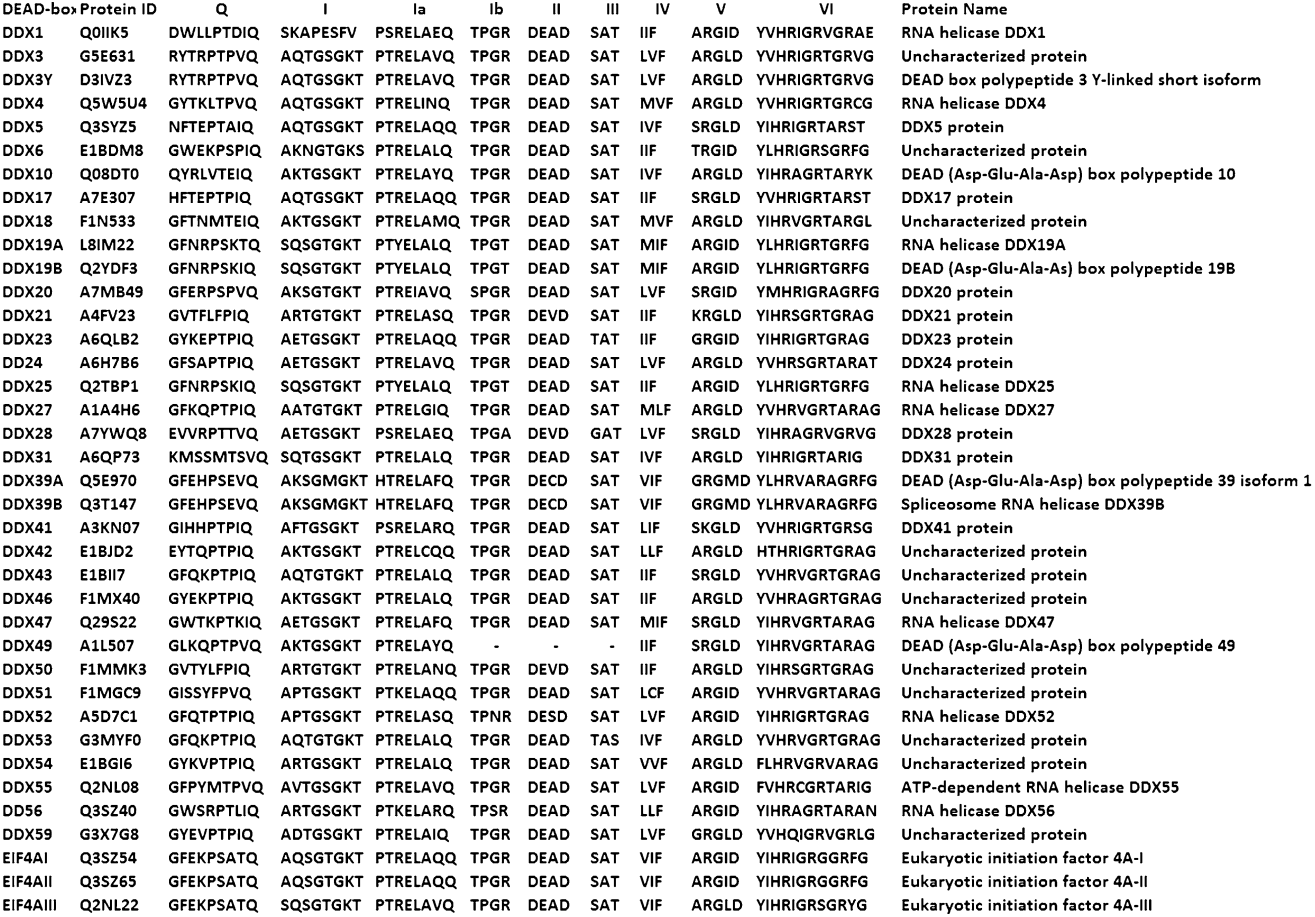
The amino acid sequence of conserved motifs constituting the RNA helicases of bovine DDX proteins

**Fig. 2:**
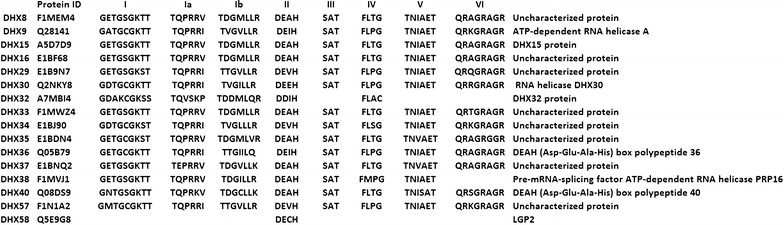
The amino acid sequence of conserved motifs constituting the RNA helicases of bovine DHX proteins

**Fig. 3:**
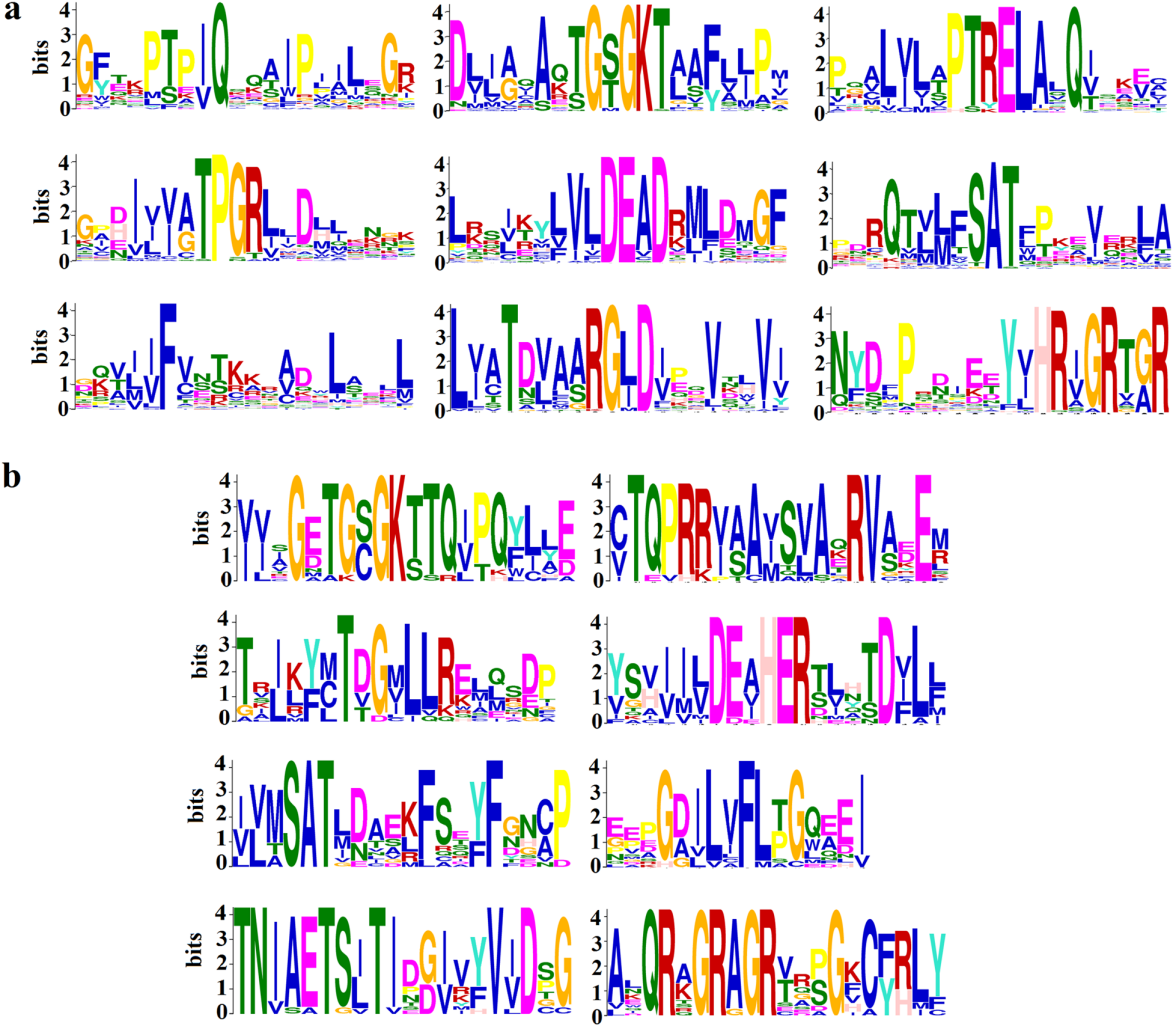
The schematic diagram of motifs of DExH/D helicases. **a** and **b** represent motifs for bovine DEAD and DEAH proteins respectively. The schematic diagrams were derived from MEME suite and generated automatically by Meme software based on scores

Genomes of all organisms have genes encoding RNA helicases. Although various comprehensive analyses of these helicases are available in various organisms, limited studies have been conducted on the role of RNA helicases in cattle. The studies of biological function of cattle RNA helicases can unravel their roles and can help in understanding different diseases in cattle and also help in improving economically important traits. Fifty four DExH/D family members of RNA helicases were identified in *Bos taurus* in the present study, amongst which 38 members belonged to DDX family (DEAD) (Table 1) and 16 members to DHX family (DEAH) of RNA helicases (Table 2). Further analysis of cattle helicase sequences with MEME suite suggested the pattern of amino acids occurrence in signature motifs validating the protein family members. Besides characteristic residues of motifs, some residues were found to be conserved around each motif of various DExH/D family members. The 38 bovine DDX members identified were DDX1, DDX3X, DDX3Y, DDX4, DDX5, DDX6, DDX10, DDX17, DDX18, DDX19A, DDX19B, DDX20, DDX21, DDX23, DDX24, DDX25, DDX27, DDX28, DDX31, DDX39A, DDX39B, DDX41, DDX42, DDX43, DDX46, DDX47, DDX49, DDX50, DDX51, DDX52, DDX53, DDX54, DDX55, DDX56, DDX59, eIF4AI, eIF4AII and eIF4AIII (Table 1). In all, 9 motifs (Q, I, Ia, Ib, II, III, IV, V and VI) were identified in these proteins which are shown in Fig. 1. The signature motifs in DDX protein showed consensus sequences as GFxxPxxIQ (Q), AxxGxGKT (I), PTRELA (Ia), TPGR (Ib), DExD (II), SAT (III), FVxT (IV), RGxD (V) and HRxGRxxR (VI). In the case of DDX49 three motifs namely; TPGR, DExD and SAT were found missing (Fig. 1). The 16 DHX members that could be identified were DHX8, DHX9, DHX15, DHX16, DHX29, DHX30, DHX32, DHX33, DHX34, DHX35, DHX36, DHX37, DHX38, DHX40, DHX57 and DHX58 (Fig. 2). Consensus sequences GxxGxGKT (I), TQPRRV (Ia), TDGML (Ib), DExH (II), SAT (III), FLTG (IV), TNIAET (V) and QRxGRAGR (VI) were found in the members of DHX proteins. Some motifs in two DHX members i.e. DHX32 and DHX58 were not found (Fig. 2). In protein DHX32, SAT, TNIAET and QRxGRAGR motifs were absent, and instead of motif DExH; DDIH motif was observed. In DHX58 conserved motif DECH was observed and remaining motifs were missing. QRxGRAGR motif was not observed in the DHX38 protein (Fig. 2). Four members i.e. DHX32, DHX58, DHX38, and DDX49 showed variable conserved motifs and need biochemical evidence for confirmation. Figure 3 describes patterns in different motifs of DDX and DHX helicases using Hidden Markov Model (HMM). In Fig 3a, b position specific probability is represented by the size of particular amino acid residue in different motifs, larger the size more will be probability of occurrence.

**Table 1:** Summary of the features of the Bovine DDX member proteins

Bos Taurus	Human	Isoelectric point	Molecular weight (kDa)	Localization	% Coverage with human	% Identity with human
DDX1	DDX1	7.23	82.43	C,N	100	97
DDX3X	DDX3X	7.2	73.15	N	100	99
DDX3Y	DDX3Y Isoform2	7.39	73.17	N	100	91
DDX4	DDX4 Isoform1	5.96	79.46	N,C	100	91
DDX5	Dead box polypeptide 5	9.21	69.16	N	100	100
DDX6	DDX6	8.93	54.39	N	99	99
DDX10	DDX10	9.17	101.18	N	100	89
DDX17	DDX17 Isoform1	8.75	72.33	N,C	100	99
DDX18X1	DDX18	10.04	75.13	N,M	100	90
DDX19A	DDX19A	6.72	54.00	C,N,	100	97
DDX19B	DDX19B Isoform1	8.54	54.46	M,N,C	95	98
DDX20	Dead box polypeptide 20	6.77	92.71	N,C	100	88
DDX23	DDX23	10.22	95.67	N	100	99
DDX24	DDX24	10.01	94.53	N	100	81
DDX25	DDX25	6.33	54.63	C,N	100	93
DDX27	DDX27	9.89	87.10	N	100	95
DDX28	DDX28	10.75	60.02	M,C,N	99	85
DDX31	DDX31	10.43	80.87	N	99	79
DDX39A	DDX39A	5.39	49.15	C,N	100	96
DDX39B	DDX39B	5.38	48.97	C,N	100	99
DDX41	DDX41	6.94	69.83	C,N,M	100	99
DDX42	DDX42	7.28	107.56	N,C	96	95
DDX43	Dead box polypeptide 43	8.77	72.04	N	99	76
DDX46	DDX46 IsoformX1	9.87	117.46	N,C	100	99
DDX47	DDX47 IsoformX1	9.64	50.92	N	100	96
DDX49	DDX49	9.82	44.39	C,N,M	99	91
DDX50	Dead box polypeptide 50	9.64	82.60	N,C	100	97
DDX51	DDX51	7.56	60.69	N,C	98	82
DDX52	DDX52	10.32	67.52	N,C	100	91
DDX53	DDX53	9.88	68.47	N	99	68
DDX54	DDX54	10.68	102.72	N	94	90
DDX55	DDX55	9.83	68.61	N,C	100	94
DDX56	DDX56 Isoform1	9.02	61.27	N,C,M	100	93
DDX59	DDX59	8.03	67.45	N,C	100	77
EIF4AI	EIF4AI Isoform1	5.12	46.15	N	100	100
EIF4AII	EIF4AII	5.13	46.41	N	100	100
EIF4A-III	EIF4A-III	6.69	46.85	N,M	100	99
Nucleolar RNA Hel2	Isoform1(DDX21)	9.87	87.25	N,C	100	89

*N, M and C* represent Nuclear, Mitochondrial and Cytoplasmic localization, respectively

**Table 2:** Summary of the features of the Bovine DHX member proteins

*Bos Taurus*	Human	Isoelectric Point	Molecular weight (kDa)	Localization	% Coverage with human	% Identity with human
DHX8	DHX8	8.33	140.28	N	99	98
DHX9	Helicase A	6.88	141.97	N	90	95
DHX15	DHX15	7.48	90.95	N	100	99
DHX16	DHX16 Iso1	6.39	119.88	N,C	100	98
DHX29	DHX29	8.67	155.28	N	99	93
DHX30	DHX30 Iso1	8.61	135.97	M,C,N	100	97
DHX32	DHX32	4.79	83.88	C,N	100	89
DHX33	DHX32 Iso1	9.23	79.75	N,C	98	92
DHX34	DHX34	7.96	128.80	N,C	100	88
DHX35	DHX35 Iso1	8.66	78.89	N	99	96
DHX36	DHX36 Iso1	7.87	114.85	N,M	100	92
DHX37	DHX37	8.93	129.02	N,C,M	100	85
DHX38	PRP16	6.55	140.19	N	100	95
DHX40	DHX40 Iso1	8.83	88.52	N,C	100	99
DHX57	DHX57	7.69	155.76	N,C	96	91
DHX58	DHX58	8.63	77.19	C,N	100	83

*N, M and C* represent Nuclear, Mitochondrial and Cytoplasmic localization, respectively

### Phylogenetic analysis

**Fig. 4:**
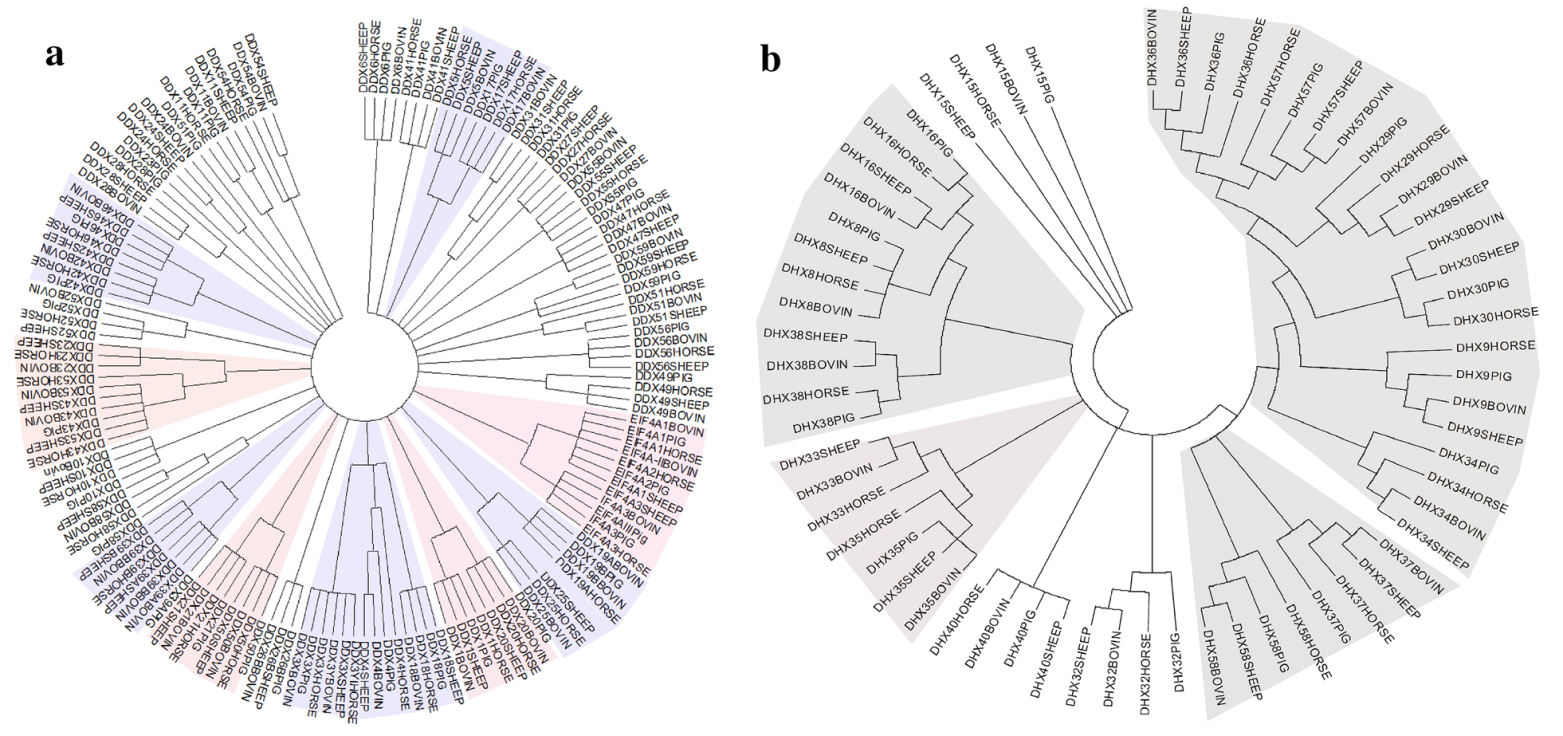
Phylogenetic analysis of RNA helicases from cattle, pig, horse and sheep. **a** and **b** represent DEAD box and DEAH box helicases from four species respectively. DEAD and DEAH amino acid sequences were aligned with ClustalW, and phylogenetic tree was constructed using the neighbour joining method in MEGA 5.0 software

**Fig. 5:**
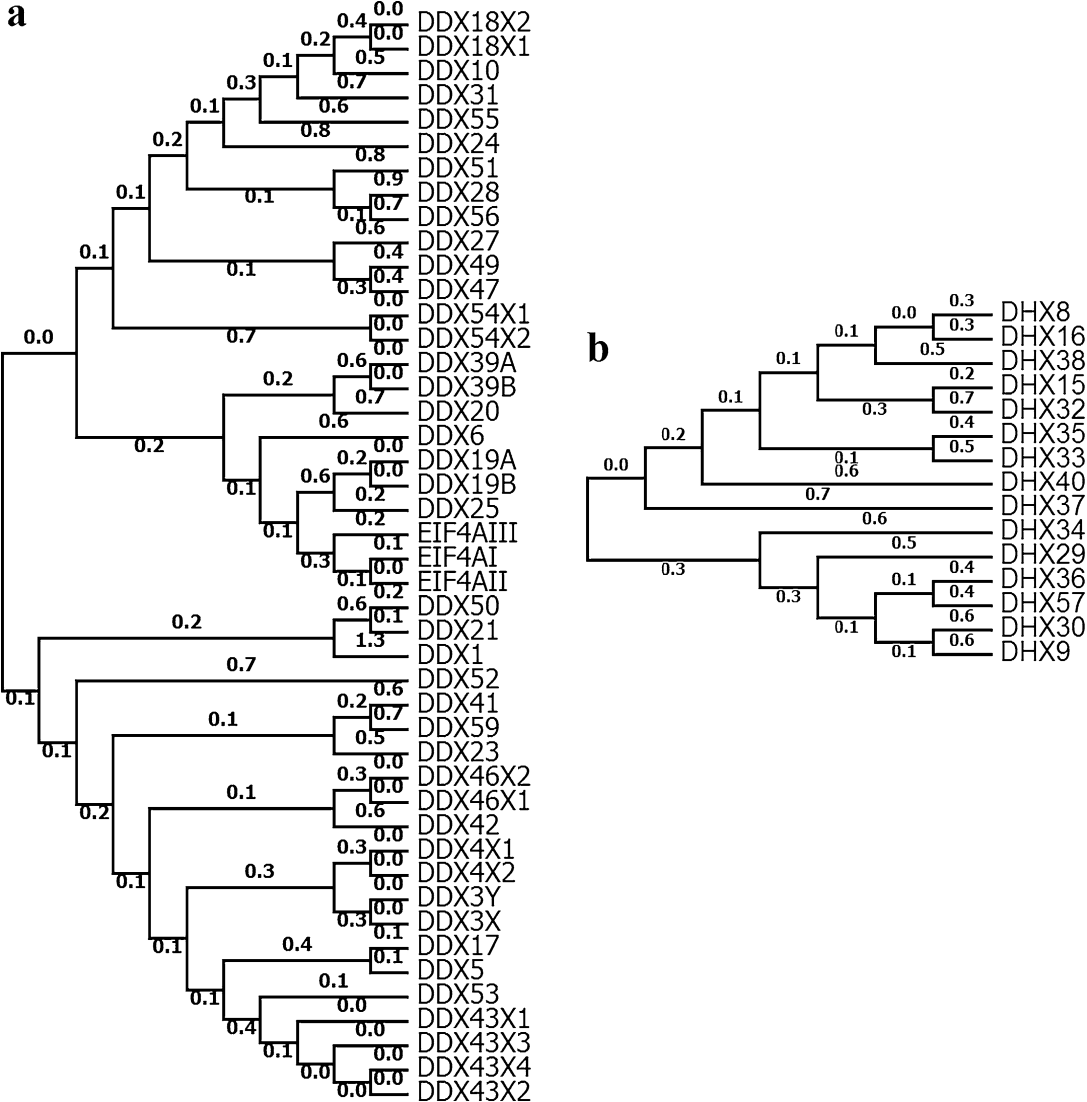
Phylogenetic analysis of Bovine DExH/D helicases. **a** and **b** represent analysis of bovine DEAD and DEAH respectively

Phylogenetic analysis of DExH/D helicases was performed to elucidate evolutionary relationship. On analysing bovine helicase with that of horse, pig and sheep (Fig. 4a, b) it was observed that some DEAD box helicase family members could be subdivided into nine subgroups in all the species taken into consideration. However, DDX 6, DDX 10, DDX 11, DDX 24, DDX 26, DDX 27, DDX28, DDX 31, DDX 41, DDX 47, DDX49, DDX 51, DDX52, DDX 54, DDX 55, DDX 56, DDX58 and DDX 59 members of DEAD box of all these species could not be included in above nine subgroups (Fig. 4a). Similarly, DHX family members could also be subdivided into four subgroups for all the species (Fig. 4b). However, DHX15, DHX32 and DHX40 could not be included in the any of these four subgroups (Fig. 4b). The extent of similarity indicates toward conserved structure of DExH/D helicases in all the species studied during evolution but their functions remained to be defined by biochemical analysis. In second analysis, relationship amongst bovine helicases was carried out (Fig. 5a, b for DDX and DHX respectively). Phylogenetic analysis established close relationship between different members. The closely related members within DDX subfamily are DDX17-DDX5, DDX43-DDX53, DDX42-DDX46, DDX4-DDX3X-DDX3Y, DDX41-DDX59, DDX39A-DDX39B, DDX19A-DDX19B, EIF4A members, DDX10-DDX18, DDX56-DDX51, DDX47-DDX49, DDX27-DDX54 and DDX50-DDX21. Similarly, within DHX members DHX8-DHX16, DHX33-DHX35, DHX15-DHX32 and DHX36-DHX57 show close relationship. All these members occur as separate clades.

### In Silico Characterization of Bovine DExH/D family members

Putative molecular weights and isoelectric points of bovine DExH helicases were determined in silico (Tables 1 and 2). Similarly predictive subcellular localizations of these proteins were examined (Tables 1 and 2). These helicases varied in their isoelectric point and molecular subunit mass. Isoelectric point of DDX members varied from 5.12 (EIF4AI) to 10.68 (DDX54) whereas pI for DHX members ranged between 4.79 (DHX32) and 9.23 (DHX33). 24 DDX and 8 DHX members had pI above 8. Molecular mass for these helicases ranged between 44.39 kDa (DDX49) and 117.46 kDa (DDX46) in case of DDX members and between 77.19 kDa (DHX58) and 155.76 kDa (DHX57) for DHX members. The predictive pI value and molecular mass will help in isolation and purification leading to further characterization of these helicases. Analysis with WoLF PSORT program indicated that cattle RNA helicases are localized in the nucleus, cytoplasm and mitochondria (Tables 1 and 2).

### Comparative analysis of human and bovine DExH/D family members and putative function assignment

**Fig. 6:**
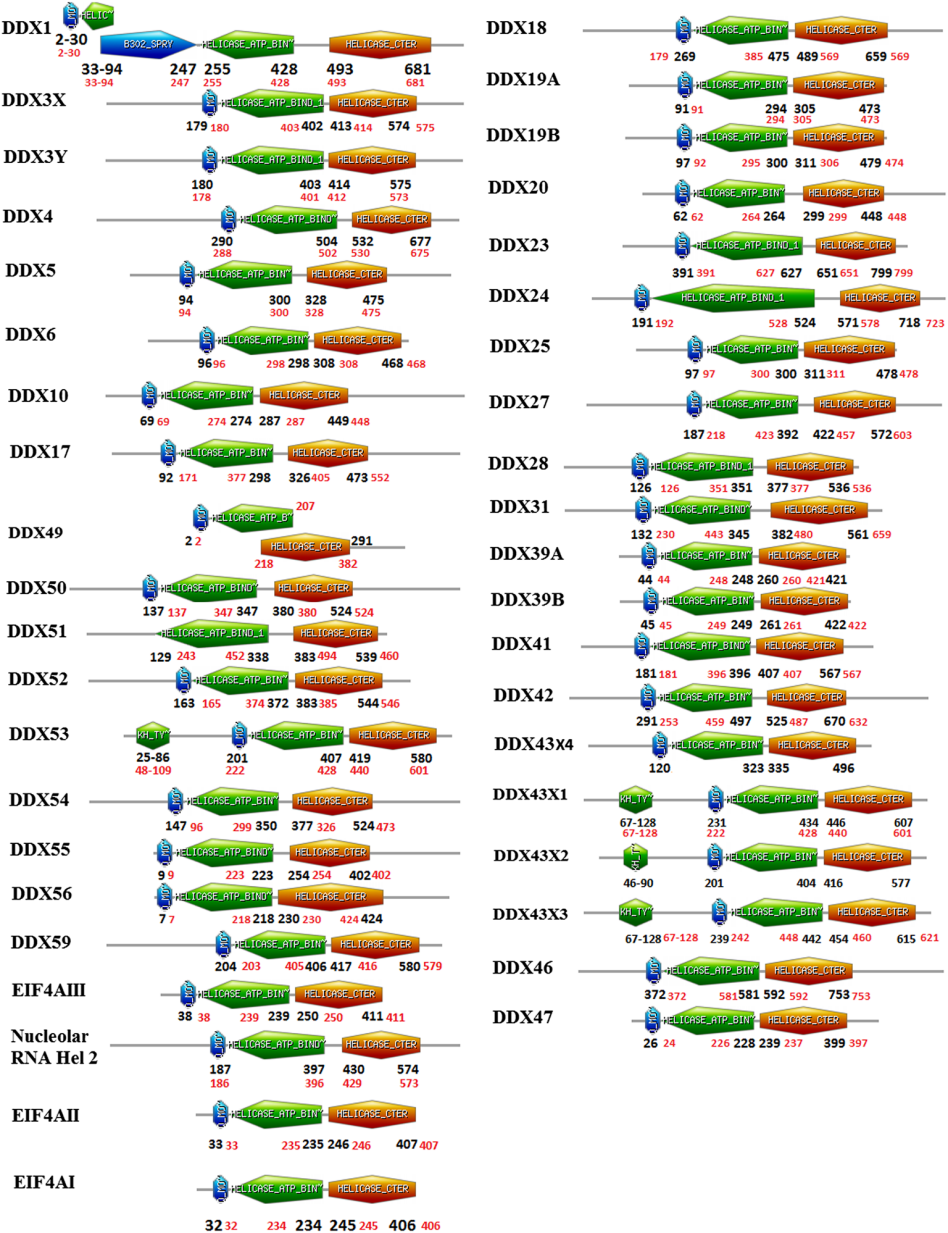
Schematic diagrams of domain organisation in bovine DEAD helicases. Domain analysis was conducted using Scan Prosite (http://expasy.org). The domain structures were downloaded and used for figure generations. The number shown in *black* and *red* colour indicates the amino acids spanning motifs in bovine and Human DEAD box proteins

**Fig. 7:**
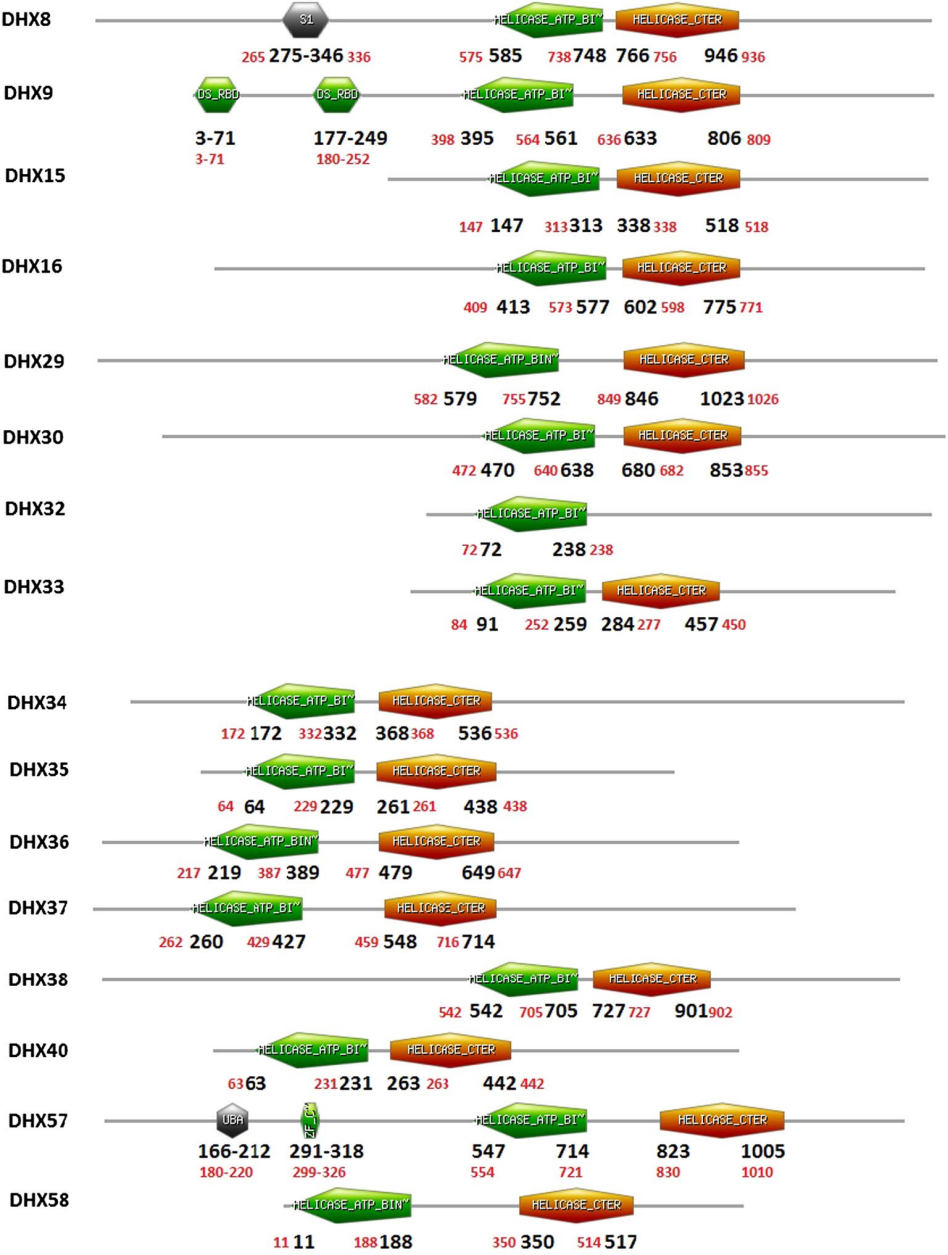
Schematic diagrams of domain organisation in bovine DEAH helicases. Domain analysis was conducted using Scan Prosite (http://expasy.org). The domain structures were downloaded and used for figure generations. The number shown in *black* and *red* colour indicates the amino acids spanning motifs in bovine and Human DEAH box proteins

*Bos taurus* has a 2.86 billion bp long genome with a minimum of 22,000 genes (The Bovine Genome Sequencing and Analysis Consortium 2009). Similarly, 2.91 billion bp long human genome has around 20,000–25,000 genes (International Human Genome Sequencing C 2004). Cattle genome encodes all orthologs of human DExH/D family members. Bovine DEAD box RNA helicases has typically Q motif, ATP binding and Helicase C-terminal domains as found in human helicases. Domain structures of bovine DExH/D RNA helicases as compared with that of human helicases indicated high similarity between the two species (Figs. 6 and 7). Despite this identity DDX17, DDX18, DDX24, DDX27, DDX31, DDX42, DDX49, DDX51, DDX53 and DDX54 show difference in positions of domains as compared to human helicases (Fig. 6). In bovine DDX49 typically overlapping of ATP binding and Helicase domain was observed. Interestingly, both bovine and human DHX32 showed only ATP binding domain and no other domain was observed. Further, levels of homology amongst human and bovine DExH/D RNA helicases are shown in Tables 1 and 2. Bovine DEAD helicases showed high similarity with their human counterpart (identity 76–100 %).

The higher similarity of these bovine helicases with well characterized human helicases can help to predict their functions in cattle developmental processes also. The putative functions of these helicases have been summarized in Tables 3 and 4. The importance of DExH/D RNA helicases in environmental stress is becoming evident (Shih and Lee 2014). DDX1, 3, 5, 6, 17, 21, 24, 47, DHX9 and DHX36 are associated with various viral infections. Similarly DDX6 and DDX19 are associated with neurological disorders, as summarised previously (Steimer and Klostermeier 2012). This manuscript presents first report on genome-wide comprehensive analysis of bovine DExH/D helicases providing valuable information regarding classification and putative function of these RNA helicases, essential for growth and development. Identification of bovine counterparts of helicases associated with various stress and diseases can be exploited as prognostic and diagnostic markers.

**Table 3:** Putative functions of DDX members

Protein	Function	Ref.
DDX1	Associated with ARE mediated mRNA decay	Chou et al. (2013)
DDX3X, DDX3Y	DDX3X can bind with DNA, RNA splicing, nuclear transport of RNA and translational regulation	Franca et al. (2007); Rosner and Rinkevich (2007)
DDX4	Bovine vasa homolog (BVH) and is expressed in gonads	Bartholomew and Parks (2007)
DDX5, DDX17	Splicing and transcriptional regulation	Auboeuf et al. (2002)
DDX6	Spermatogenesis and localized in spermatogenic cells	Kawahara et al. (2014)
DDX10	Ribosome assembly	Savitsky et al. (1996)
DDX18	Hematopoiesis and deletion resulted into p-53 depended cell arrest in G1	Payne et al. (2011)
DDX19	m-RNA nuclear transport by remodelling of RNP particles through nuclear pore complex	Collins et al. (2009)
DDX20	Transcriptional regulation, splicing process and mi-RNA pathway	Takata et al. (2012)
DDX23	Pre-mRNA splicing	Ismaïli et al. (2001)
DDX24	Innate immune signalling regulation	Ma et al. (2013)
DDX25	Posttranscriptional regulations of genes for spermatid elongation & completion of spermatogenesis	Dufau and Tsai-Morris (2007)
DDX27	ND	
DDX28	Cellular division	Loo et al. (2012)
DDX31	Transcription of rRNA gene and assembly of 60 s ribosomal subunit	Bish and Vogel (2014)
DDX39	mRNA splicing, genome integrity and telomere protection	Yoo and Chung (2011)
DDX41	Type 1 interferon response	Zhang et al. (2011a)
DDX42	Function as chaperon	Uhlmann-Schiffler et al. (2006)
DDX43	ND	
DDX46	Pre-mRNA splicing	Hozumi et al. (2012)
DDX47	Pre-RNA processing	Sekiguchi et al. (2006)
DDX49	ND	
DDX51	Ribosome synthesis and formation of 3′end of 28S rRNA	Srivastava et al. (2010)
DDX52	ND	
DDX53	ND	
DDX54	Maintenance of central nervous system	Zhan et al. (2013)
DDX55	ND	
DDX56	Assembly of pre-ribosomal particles	Zirwes et al. (2000)
DDX59	Pathogenesis of orofaciodigital syndrome	Shamseldin et al. (2013)
EIF4A	eIF4F complex formation and facilitates translation	Harms et al. (2014)
Nucleolar RNA Hel2 (DDX21)	RNA processing during interphase of mitosis	De Wever et al. (2012)

**Table 4:** Putative functions of DHX members

Protein	Function	Ref.
DHX8	Mitosis and involved in mRNA splicing	English et al. (2012)
DHX9	RNA induced silencing complex (RISC) loading factor	Fu and Yuan (2013)
DHX15	RNA virus sensing and activating immune system	Lu et al. (2014)
DHX16	Splicing	Gencheva et al. (2010)
DHX29	Protein synthesis	Pisareva et al. (2008)
DHX30	Mitochondrial DNA replication	Zhou et al. (2008)
DHX32	Lymphocyte differentiation and T cell apoptosis	Huang et al. (2009)
DHX33	rRNA transcript and nucleolar organizer	Zhang et al. (2011b)
DHX34	NMD (nonsense-mediated mRNA decay)	Anastasaki et al. (2011)
DHX35	ND	
DHX36	Viral nucleic acid sensors, affinity towards G4-quadruplex	Fullam and Schroder (2013)
DHX37	Glycinergic synaptic transmission and associated motor behaviour	Hirata et al. (2013)
DHX38	Associated with retinitis pigmentosa	Ajmal et al. (2014)
DHX40	Pre mRNA splicing and ribosome biogenesis	Xu et al. (2002)
DHX57	ND	
DHX58	Innate antiviral immune response	Li et al. (2009)

## Conclusions

*Bos taurus* genome encodes 54 DExH/D family members (38 DDX and 16 DHX). Present work describes their evolutionary relationship, putative functions, pI, molecular weight and localization. Despite high similarity with well characterized counterparts, for some members, functions could not be predicted which needs further analysis. Hence, this study emphasises towards some bovine DExH/D members requiring further biological characterisation. Similarly, bovine DDX49 and DHX32 need biochemical characterization as they showed unique properties. Association analysis of these members with different abiotic and biotic stress may facilitate new diagnostic markers and drug targets.
